# Weight-inclusive intuitive eating course associated with reductions in disordered eating in adults: a mixed methods study

**DOI:** 10.3389/fpsyg.2026.1953974

**Published:** 2026-10-06

**Authors:** Nikole Decker Squires, Dawn Clifford, Sarah Mensah Asantewaa, Stephanie Garrison, Nick Galli

**Affiliations:** 1 Department of Health Sciences, Northern Arizona University, Flagstaff, AZ, United States; 2 Department of Physical Therapy, Northern Arizona University, Flagstaff, AZ, United States; 3 Department of Atheletics, University of Utah Health, Salt Lake City, UT, United States

**Keywords:** body image, disordered eating, health promotion, intuitive eating, weight bias, weight-inclusive

## Abstract

**Introduction:**

Many employee health promotion programs encourage lifestyle changes such as eating more fruits and vegetables or getting more physical activity. Most of these programs operate from a weight-normative lens, meaning they encourage such changes with the primary goal of weight change (typically weight loss). However, research indicates that weight-inclusive programs - programs that focus on health behaviors alone, regardless of weight change - may be just as effective at promoting better health and also feel less stigmatizing for participants. The purpose of this study was to test the effects of a weight-inclusive employee wellness program (Intuitive Eating, or IE) and a weight-normative program (Intensive Lifestyle Program, or ILP) on disordered eating, intuitive eating, weight bias internalization, weight bias, weight/shape concerns, and body appreciation of adults working in a university setting.

**Methods:**

This study utilized a quasi-experimental, mixed-methods approach and employed 8- (IE) and 12-week (ILP) interventions with validated pre and post questionnaires and semi-structured interviews. Quantitative data was analyzed using two-way repeated measures ANOVAs and qualitative data was analyzed using interpretive phenomenological analysis (IPA).

**Results:**

Participants (*n* = 42) were 95% white and 98% female. 26 in the IE group and 16 in the ILP group enrolled in the study. Of that sample, 13 participants were interviewed (IE = 6; ILP = 7). The IE program was associated with significantly greater reductions in disordered eating from pre- to post-course compared to the ILP program. There were no statistically significant improvements in weight/shape concerns, IE levels, body appreciation, and weight bias between groups from pre- to post-course. Qualitative analysis found 5 main themes: 1) Influences on Body Image, 2) Diet Culture, 3) Forms of Eating and Exercise, 4) Mental and Emotional Wellbeing, and 5) Self-Care.

**Discussion:**

Qualitative analysis provided richer detail from in-depth individual perspectives of participants’ attitudes in both groups about eating attitudes/behaviors, body image, and weight bias. Employee wellness programs grounded in weight-inclusive approaches such as intuitive eating may be associated with reductions in disordered eating and provide a non-stigmatizing approach to health promotion in a homogenous employee wellness setting.

## Introduction

Many worksite wellness programs focus on weight loss or weight maintenance to improve health, despite mounting evidence of probable weight regain (Jane et al., 2018; Jones et al., 2019; Kahathuduwa et al., 2016; Lavie et al., 2018; Mattke et al., 2013; Rothblum, 2018; The Diabetes Prevention Program (DPP) Research Group, 2002). Weight-inclusive health promotion programs provide an alternative approach, focusing on health-supporting behaviors regardless of weight changes (Mauldin et al., 2022). This approach has been shown to improve both physical and mental health (Bégin et al., 2019; Carbonneau et al., 2017; Clifford et al., 2015; Schaefer and Magnuson, 2014). Intuitive Eating (IE) is one weight-inclusive approach that centers on making peace with food, body and movement (Tribole and Resch, 2020; Tylka and Kroon van Diest, 2013). Intuitive eating has 10 underlying principles (see Table 1) and shows promise in preventing weight cycling, negative body image, and disordered eating (Ogden et al., 2020; Richards et al., 2017; Tylka et al., 2020).

**Table 1:** The ten principles of intuitive eating (IE) (Tribole and Resch, 2020).

IE principle	Brief definition of IE principle
Reject the Diet Mentality	Reflect on diet history and harmful weight myths. Practice taking a more flexible approach to food, body, and movement.
Honor Your Hunger	Get in touch with physiological hunger cues and differences between emotional and biological hunger. Practice eating when physically hungry.
Make Peace with Food	Give oneself unconditional permission to eat any and all foods, nutrient-dense or otherwise.
Challenge the Food Police	Practice talking back to unhelpful, negative thinking around food. Engage in self-compassion, mindfulness, and cognitive restructuring/reframing techniques. Set boundaries with people who may police food/body.
Discover the Satisfaction Factor	Learn about the importance of satisfaction and pleasure in eating. Satisfaction is the “hub” and focus of intuitive eating.
Feel Your Fullness	Get in touch with physiological fullness/satiety cues and differences between fullness and guilt/shame.
Cope with Your Emotions with Kindness	Check in with ways one identifies and handles difficult emotions. Learn how to discover unmet wants and needs through emotions. Expand coping skills to work through tough emotions.
Respect Your Body	Introduce body appreciation and its tenets of respect, self-compassion, and functionality.
Movement—Feel the Difference	Consider barriers to movement related to diet mentality. Discuss ways to incorporate joyful, mindful movement into daily life.
Honor Your Health with Gentle Nutrition	Learn basic tenets of nutrition around variety, balance, and moderation. Relate gentle nutrition back to rejecting diet mentality

While research supports IE-based interventions, few studies have evaluated interventions that included all ten IE principles (Cole and Horacek, 2010; Burnette et al., 2022; Burnette and Mazzeo, 2020). Those that did demonstrated promise in reducing disordered eating, body image, and weight bias internalization, but lacked in-depth participant experiences or comparisons to weight-normative programs (Cole and Horacek, 2010; Burnette et al., 2022; Burnette and Mazzeo, 2020), indicating the need for more research.

The purpose of this research was to examine the effects of an 8-week IE worksite wellness intervention on body image, weight bias, and disordered eating patterns of adults (>18 and <65 years old) working on a university campus. We hypothesized that participants in the weight-inclusive, Intuitive Eating program would decrease disordered eating and internalized weight bias and improve body image more than participants in the weight-normative comparison group.

## Materials and methods

This project utilized a quasi-experimental, explanatory sequential design with validated questionnaires and semi-structured interviews. A mixed-methods approach was chosen to showcase a unique perspective regarding physical, mental, and emotional effects of weight-inclusive and weight-normative health promotion programming.

### Health promotion program descriptions and intervention/program design

Participants in the weight-inclusive intervention, “Intuitive Eating: Making Peace with Food, Body, and Movement” (WI-IE) were compared to those in the weight-normative program, “Intensive Lifestyle Program” (WN-ILP). Both were employee wellness programs that were held virtually via Zoom at a large, public southwestern university in spring 2022. Employees could receive discounts on their insurance premiums for participating in either program. Participants were recruited to the study after they signed up for either the WI-IE or WN-ILP programs.

There were three 8-week IE intervention courses and two 12-week ILP courses held at varying days and times to allow participants with a wide variety of schedules to participate. Both groups had trained facilitators in their respective programs. Surveys were distributed before the first week and 8 weeks into the IE and ILP courses, respectively.

*Weight-Inclusive IE Intervention Program (WI-IE)*. The 8-week WI-IE course was based on both the 4th edition of the *Intuitive Eating* book (Tribole and Resch, 2020) and the 1st edition of the *Intuitive Eating Workbook* (Tribole and Resch, 2017). This study was conducted before the second edition of the *Intuitive Eating Workbook* was released. Weekly program sessions were 60-75-min. The first 5–10 min of each class included a brief mindfulness activity and sharing time around challenges and successes implementing IE principles. The remaining time included an education portion on one of the 10 IE principles (Table 1) with accompanying questions, activities, and discussion to facilitate active learning and application of each principle. Before and after class each week, WI-IE participants had the opportunity to access optional supplemental readings and activities through a Canvas course.

The 8-week course taught the IE principles in the following order: (1) Introduction to Intuitive Eating; (2) Reject the Diet Mentality + Honor Your Hunger; (3) Make Peace with Food + Challenge the Food Police; (4) Discover the Satisfaction Factor + Feel Your Fullness; (5) Cope with Your Emotions with Kindness; (6) Respect Your Body; (7) Movement— Feel the Difference; and (8) Honor Your Health with Gentle Nutrition.

*Weight-Normative ILP Program (WN-ILP)*. The 12-week WN-ILP course included weekly 90-min sessions on general healthy lifestyle principles such as meal planning, exercise, nutrition, and stress management. The first 60–70 min included a lecture and an activity or two on a health-related topic. In the remaining 20–30 min of class participants asked questions and discussed applying that week’s topic. Since the ILP program had a separate exercise prescription component, participants were screened for heart disease and required clearance from a physician to participate in physical activity outside of class sessions. While the program was not advertised explicitly as a weight-loss program, many participants joined for the purpose of losing weight in tandem with other health goals such as increasing physical activity levels or better controlling blood glucose levels. Participants were encouraged to engage in behavior change goals they set at the beginning of the program.

The 12-week course taught general healthy lifestyle principles in the following order: (1) Orientation, Monitoring Health, Tracking; (2) Whole Person Wellness; (3) Exercise (Guest Speaker); (4) Nutrition 101 Basics; (5) Food and Mood & Nutrition Labels; (6) Meal Planning; (7) Intuitive Eating; (8) ACT (Guest Speaker); (9) PERMA (Guest Speaker); (10); Coping with Challenging Emotions; (11) Managing Stress; and (12) Staying Motivated. In addition, participants were encouraged to track calories/macros and activity levels during the Monitoring Health and Tracking segment. The WN-ILP program also included a short segment on intuitive eating as a strategy for improving health and weight management.

### Participants

Researchers recruited participants for the two programs through word-of-mouth and a large, university-wide email distributed by the hosting employee wellness office. Program participants were invited to participate in the research study when they began their respective programs. This research was approved by the Institutional Review Board (IRB) at a mid-sized university in the southwest. Consenting participants completed the pre-screening questions with inclusion and exclusion criteria and were invited to sign up for an additional 15-30-min one-on-one interview with a member of the research team. Participants who met study criteria were also sent the pre-course survey prior to the start of the program. Post-surveys were distributed 8 weeks into each program, which left the WN-ILP program with 4 remaining weeks. However, this time-frame for “post” survey distribution was carefully chosen to give each group the same dose of their respective course, despite varying course lengths. Participation incentives for surveys and interviews included pre- and post-course gift certificates of $25 each. Please see Figure 1 for the study recruitment process and attrition details.

**Figure 1:**
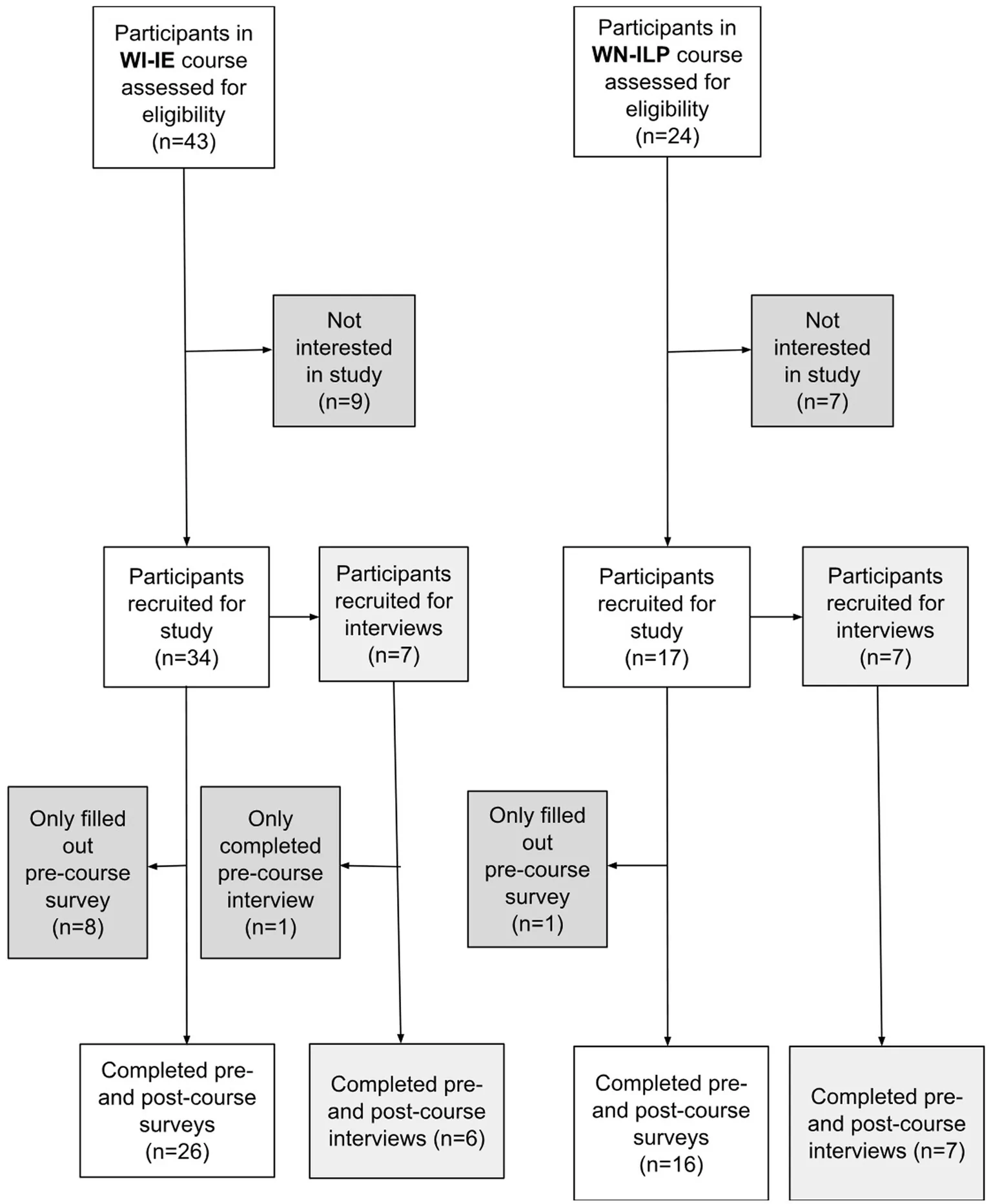
Participant recruitment and study process.

### Measures

*Surveys*. The survey was completed on REDCap (Research Electronic Data Capture; Harris et al., 2019), took approximately 15–20 min to complete, and had 133 items in total. Participants created a unique number identifier to allow for matching pre- and post-intervention data. Demographic questions included race, age, gender, height, weight, job title, education level, and dieting history. Measures assessed disordered eating, weight bias internalization, and body image. For all measures below, higher scores indicate higher levels (e.g., of disordered eating, of intuitive eating, etc.).

*Disordered Eating*. Disordered eating behaviors and attitudes were measured using the global score from the 28-item Eating Disorder Examination Questionnaire (EDE-Q; Jennings and Phillips, 2017), which uses 7-point Likert scale items to measure behavior frequency (No days to Every day).

*Intuitive Eating*. Intuitive eating levels were measured utilizing the total score from the Intuitive Eating Scale-2 (IES-2; Augustus-Horvath and Tylka, 2011). The IES-2 is a 23-item questionnaire with 5-item Likert scale questions measuring agreement (Strongly disagree to Strongly agree). The IES-3 (Tylka et al., 2024) was not used because it had not yet been validated when this research was conducted.

*Body Image*. Body image was measured by examining body appreciation and body dissatisfaction. Body appreciation was measured using the Body Appreciation Scale-2 (BAS-2; Tylka and Wood-Barcalow, 2015). The BAS-2 is 10 items long and includes 5-item Likert-type scale questions to measure frequency of events (never to always). Body dissatisfaction was measured using the Weight Concerns Subscale and the Shape Concerns Subscale from the EDE-Q.

*Weight Bias Internalization*. Weight bias internalization (WBI) was measured using the Modified Weight Bias Internalization Scale (WBIS-M; Pearl and Puhl, 2014, 2018). The WBIS-M was designed to measure WBI in people of all weight ranges. The WBIS-M is 11 items with 7-item Likert scale questions to measure agreement (Strongly disagree to Strongly agree).

*Weight Bias*. The Anti-Fat Attitudes Scale-Revised (AFAS-R) is a 24-item questionnaire measuring general anti-fat attitudes, with 5-item Likert scale questions to measure agreement (Strongly disagree to Strongly agree; Wrench and Knapp, 2008).

For all instruments above, higher scores indicate higher levels. All Cronbach’s alpha internal reliability measures were >0.80 pre- and post-course except for the IES-2, which was 0.60 at pre-course.

*Semi-Structured Interviews*. Individual 15-30-min interviews were conducted via Zoom 1 week pre-intervention and at post-intervention. Interviews—guided by DeJonckheere and Vaughn’s (2019) principles—explored participants’ relationships with food, exercise, and body image. They were recorded and followed a predetermined question guide with possible probes (see Appendix A).

### Data analysis

*Surveys*. Deidentified data from the surveys was exported into the Statistical Package for the Social Sciences (SPSS), version 28.0 (Field, 2018). Data analyzers ran missing data patterns (*p* = 0.999), normality, frequency tables, and descriptive statistics on demographic variables to create means and standard deviations for the WI-IE and WN-ILP groups. Finally, our team ran repeated measures two-way ANOVA analyses on the appropriate scales and subscales of the survey to determine if results showed moderate-to-strong effects in the WI-IE group versus the WN-ILP group pre- and post-course. We conducted a Bonferroni correction to correct for family-wise errors and inflated alpha, type 1 error, and family-wise error (Armstrong, 2014). Thus, the Bonferroni correction test changed the level of significance from α < 0.05 to α < 0.007 (0.05/7 tests = 0.0071). Confidence intervals were set to 95% and α to 0.007 for significance level. Effect sizes for the ANOVA tests were categorized as a large effect size ≥ 0.14; medium effect size ≥ 0.06; and small effect size ≥ 0.01, per Adams and Conway (2014).

*Interviews*. Interviews were transcribed using Otter.ai and reviewed for accuracy. Three graduate students in health-related fields independently coded the transcripts and collaboratively identified overarching themes, ensuring triangulation and consistency. Before coding the transcripts, the coders also wrote positionality statements to become aware of any potential personal or professional biases.

Interpretive Phenomenological Analysis (IPA) was chosen to analyze the interviews to highlight how participants subjectively “made meaning” of their experiences within the context of their worldviews (Pietkiewicz and Smith, 2014; Smith et al., 2022). Coders first documented emotional responses, initial impressions, and concrete meanings (Smith et al., 2022). The coders expanded upon initial codes with ideas, concepts, or issues raised in the interview that aligned with or diverged from the quantitative data results. These expansions were utilized to transform initial codes into major themes related to body image, disordered eating, and weight bias (general and internalized). The coders met to agree upon finalized themes and subthemes that both reflected interviewees’ lived experiences and expanded upon possible reasons for quantitative data results. Theme saturation was met for all themes and subthemes (Saunders et al., 2018).

## Quantitative results

Of the 43 WI-IE participants who started the course, 41 completed the course and 34 enrolled in the study. However, only 26 completed both pre- and post-course surveys. Similarly, 22 of 24 WN-ILP participants who started the course, completed the course and 17 enrolled in the study. Only 16 completed pre- and post-course surveys (see Figure 1). There were no significant differences in study completion rates or in study completers and non-completers at baseline between groups.

Participants in both groups were primarily white (95%) females (98%) with at least an undergraduate degree (72%). Participants in both groups were on average 42 years old and had an average body mass index (BMI) of 31.55. Sixty-seven percent of participants had a history of dieting (started a diet at some point in their lives with the intention to change body weight and/or shape).

These samples held enough power to identify medium and large between- and within-group effects over two timepoints (Faul et al., 2009). There were no significant differences in demographic variables between groups.

### Surveys

*Disordered Eating*. The results of the two-way repeated measures ANOVA revealed that there was a statistically significant interaction effect of the course group and time on disordered eating levels at the α < 0.007 level. Participants in the WI-IE group experienced significantly greater reductions in disordered eating levels from pre- to post-course. The WI-IE group participants experienced on average a 44.0% reduction in disordered eating levels compared to a 9.3% reduction in the WN-ILP group participants (Tables 2, 3).

**Table 2:** Means and standard deviations for the disordered eating, intuitive eating, body image, and weight bias scales from pre- to post-program.

Outcomes	WI-IE Pre *M*(SD) *n* = 26	WI-IE Post *M*(SD) *n* = 26	WN-ILP Pre *M*(SD) *n* = 16	WN-ILP Post *M*(SD) *n* = 16	Total Pre *M*(SD) *n = 42*	Total Post *M*(SD) *n* = 42
Disordered eating						
EDE-Q^a^	2.75 (1.13)	1.54 (0.77)	2.47 (1.19)	2.24 (1.17)	2.64 (1.15)	1.80 (0.99)
IES-2^b^	2.69 (1.08)	3.27 (0.83)	3.40 (0.98)	3.48 (0.71)	2.96 (1.09)	3.35 (0.83)
Body image						
BAS-2^c^	2.73 (0.86)	3.37 (0.68)	3.13 (0.71)	3.38 (0.64)	2.88 (0.82)	3.37 (0.66)
EDE-Q Shape Concern^d^	3.81 (1.42)	2.51 (1.24)	3.52 (1.56)	3.13 (1.60)	3.70 (1.47)	2.75 (1.40)
EDE-Q Weight Concern^d^	3.29 (1.35)	2.25 (1.24)	3.08 (1.46)	2.81 (1.50)	3.21 (1.38)	2.46 (1.36)
Weight bias internalization and weight bias						
WBIS-M	4.66 (1.07)	4.02 (1.50)	4.05 (1.22)	4.04 (1.57)	4.43 (1.16)	4.03 (1.51)
AFAS-R	2.06 (0.48)	1.83 (0.53)	2.00 (0.43)	1.84 (0.46)	2.03 (0.46)	1.84 (0.50)

M = mean. SD = standard deviation. n = number of people per group.

a Disordered eating measured with the Eating Disorders Examination Questionnaire (EDE-Q); total possible scores range from 1 to 7.

b Intuitive eating measured with the Intuitive Eating Scale-2 (IES-2); total possible scores range from 1 to 5.

c Body appreciation measured with the Body Appreciation Scale-2 (BAS-2); total possible scores range from 1 to 5.

d Body dissatisfaction was measured with the EDE-Q shape and weight concern subscales; total possible scores range from 1 to 7.

**Table 3:** Results from two-way, repeated measures ANOVAs for the disordered eating, intuitive eating, body image, and weight bias scales from pre- to post-program.

Outcomes	Sum of squares	df	Mean square	*F*	*p*	*η_p_^2^*
Disordered eating						
EDE-Q						
Time**	10.258	1	10.258	25.285	<0.001	0.387^a^
Group	0.914	1	0.914	0.505	0.481	0.012
Time*Group**	4.808	1	4.808	11.851	0.001	0.229
IES-2						
Time**	2.159	1	2.159	9.358	0.004	0.19
Group	4.132	1	4.132	2.67	0.11	0.063
Time*Group*	1.207	1	1.207	5.23	0.028	0.116
Body image						
BAS-2						
Time**	3.82	1	3.82	30.15	<0.001	0.43
Group	0.83	1	0.83	0.866	0.358	0.021
Time*Group*	0.76	1	0.76	5.97	0.019	0.13
EDE-Q shape concern						
Time**	14.073	1	14.073	18.994	<0.001	0.322
Group	0.527	1	0.527	0.157	0.694	0.004
Time*Group*	4.192	1	4.192	4.192	0.022	0.124
EDE-Q weight concern						
Time**	8.48	1	8.48	13.972	<0.001	0.259
Group	0.6	1	0.6	0.191	0.664	0.005
Time*Group*	3.04	1	3.04	5.01	0.031	0.111
Weight bias internalization and weight bias						
WBIS-M						
Time*	2.1	1	2.1	5.062	0.03	0.112
Group	1.77	1	1.77	0.555	461	0.014
Time*Group*	1.956	1	1.956	4.714	0.036	0.105
AFAS-R						
Time**	0.727	1	0.727	14.669	<0.001	0.268
Group	0.011	1	0.011	0.025	0.874	0.001
Time*Group	0.021	1	0.021	0.429	0.516	0.011

N = number of participants in each group; df = degrees of freedom; F = F-statistic; p = p-value; η_p_^2^ = partial eta squared; EDE-Q = Eating Disorder Examination Questionnaire; IES-2 = Intuitive Eating Scale-2; BAS-2 = Body Appreciation Scale-2; WBIS-M = Modified Weight Bias Internalization Scale; AFAS-R = Revised Anti-Fat Attitudes Scale. *p < 0.05. **p < 0.007.

a Large effect size ≥ 0.14; medium effect size ≥ 0.06; small effect size ≥ 0.01.

Mean changes in IE levels (IES-2) showed improvements in the WI-IE group from pre- to post-course compared to the WN-ILP group (Table 2). However, the interaction effect between groups pre- and post-course was not statistically significant at the α < 0.007 level. Despite not being statistically significant, there was a medium effect (Table 3).

*Body Image*. *Body Appreciation*. The WI-IE participants experienced greater increases in body appreciation (BAS-2) compared to the WN-ILP group with medium effect, although these increases were not statistically significant at the α < 0.007 level (Tables 2, 3).

*Weight and Shape Concern (Body Dissatisfaction)*. The ANOVA for the EDE-Q weight concern subscale revealed that the interaction effect across groups and time was not statistically significant at the α < 0.007 level (Table 3). However, there was a medium effect and the WI-IE group EDE-Q weight concern subscale averages dropped 1.04 points lower from pre- to post-course, compared to 0.40 points lower in the WN-ILP group (Table 2). Lastly, the EDE-Q shape concern ANOVA revealed that shape concern decreased more in the WI-IE group pre- to post-course than the WN-ILP group with medium effect, although this difference was not statistically significant at the α < 0.007 level (Table 3).

*Weight Bias Internalization*. The WI-IE group participants experienced a larger decrease in WBI (WBIS-M) from pre- to post-course than the WN-ILP group (Table 2). The ANOVA revealed that these differences were not statistically significant at the α < 0.007 level, although there was a medium effect (Table 3). As the WBI data showed non-normal, platykurtic distribution, Friedman’s test was also employed. Friedman’s test indicated similar results (*X^2^*(1) = 3.60, *p* = 0.06), and was not statistically significant at the α < 0.007 level.

*Weight Bias*. The interaction effect of overall weight bias reduction (AFAS-R) was not statistically significant at the α < 0.007 level for differences across groups and time, with small effect (Table 3).

## Qualitative results

### Interviews

Six participants from the WI-IE program and 7 from the WN-ILP program completed interviews. Five main themes emerged across both groups of participants: (1) Influences on Body Image, (2) Diet Culture, (3) Forms of Eating and Exercise, (4) Mental and Emotional Wellbeing, and (5) Self-Care. Each theme had several subthemes (Table 4) that further illustrated and colored the main theme.

**Table 4:** Themes, subthemes, and definitions found from IPA.

Theme	Definition
Influences on body image—things that have impacted a person’s body image over their lifetime	
Subthemes	
Childhood/adolescence	Weight bias/stigma
External comparison	Diet mentality (all-or-nothing)
Beauty standards	Sense of community
Societal/familial pressure	External motivation
Diet culture—reflection around scales and numbers	
Subthemes	
Traditional weight loss items	Misconceptions of intuitive eating
Diet mentality (shifting away from)	
Forms of eating and exercise—types of eating and exercise mentioned by interviewees	
Subthemes	
Disordered eating/exercise	Intuitive eating
Flexibility towards food/exercise	
Mental and emotional wellbeing—outlook around food/body/movement and its impacts on internal health	
Subthemes	
Internal comparison	Body image
Internalization of beauty standards	Shame and guilt around food/body/movement
Self-care—behaviors and thoughts related to acts of caring for oneself	
Subthemes	
Self-care behaviors/thoughts	Flexibility towards food/exercise
Healing from dieting/exercise	Self-acceptance
Body trust	Internal motivation

IPA, Interpretive Phenomenological Analysis.

All thirteen participants had coded quotes related to all five themes, though not every participant had coded quotes in every single subtheme. Subthemes were kept when at least half of participants mentioned the subtheme in their interview (Miles et al., 2020). The most common subthemes seen across all participants included themes such as childhood/adolescence impact; societal/familial pressure; shame and guilt around food/body/movement; and flexibility towards food/exercise. For specific quotes related to the themes and subthemes, see highlighted quotes in Table 5 and in the Discussion section.

**Table 5:** Example quotes to illustrate relevant themes and sample subthemes.

Theme	
Subtheme	*Quote* (Pseudonym, Program, Pre- or Post-Intervention)
Influences on body image	
Childhood/adolescence impact	*But it’s just something that kids would do: growing up, getting older in school, comparing yourself to each other. All those sorts of things I think over time – and of course, the media, whatever you see – just contributes to those thoughts of what is considered healthy, attractive versus what is not.* (Bethany, WI-IE, post-int.)
Weight bias/stigma	*Well, and even if it’s not directly aimed at me, I’ve been in areas where somebody would make a comment and it’s usually about the guys in the room or whatever being overweight or eating too much or anything like that. Even though it wasn’t directed at me, I internalize that because I know how I compare to that person, right? It’s like, “Well, if they are thinking that about him, they are obviously thinking that about me too.”* (Charlotte, WI-IE, pre-int.)
Diet culture	
Diet mentality	*With food, it’s been that I’m not restricting or anything anymore. I was doing no sugar, no flour with the last thing I was doing, and feeling very deprived. So I’m not restricting and in that sense, it’s like I said: it’s showing that I’m not binging either, but neither am I not doing without. I have better times than others, but for the most part it’s [my relationship with food] much improved all the way around.* (Nancy, WI-IE, post-int.)
Misconceptions of IE	*Well, I thought that I knew what intuitive eating was all about. I’m like, “Oh, yes, this!” But even just from our first class, we had our misconceptions [discussion] and I thought, “Oh, yeah. I guess I did fall into that.”* (Allie, WI-IE, pre-int.)
Forms of eating/exercise	
Disordered eating/exercise	*The part of food that I do not like is this hurried and rushed, unconsciously thinking, eating that I do during the day that I do not even count as eating but really is eating. And so that’s why I think that distorted eating part is for me. But it’s [when] I dropped my kids off, so 8:15 to 4:00 o’clock, that’s my disordered eating time. Or the kids have gone to bed and I’m so exhausted and now I’m grocery shopping, and I’m choosing something that feels sweet or like I’ve earned it, and then I’ll eat something that I do not necessarily need or even want. I’m not even valuing it; I’m not even appreciating what I’m eating. I’m scarfing it, so that’s one piece.* (Lilly, WN-ILP, pre-int.)
Intuitive eating	*I feel like again with food, what I’m trying to achieve is finding a way to have food that makes me feel better throughout the day and that sustains me. I feel like a lot of times I’ll just quickly eat pasta and then I’m hungry an hour later because of not eating something that’s filling me up. And so I feel, as far as quality-of-life, I’m just not eating the most nutritious and things that make me feel happy and motivated and like I want to move and do stuff.* (Stephany, WN-ILP, pre-int.)
Mental and emotional wellbeing	
Internal comparison	*A couple months ago I felt in a really good place. I had a little bit of fat still, which was fine. But then I was on a medication that helped me control my eating in a way and I got taken off of it and now I gained all this weight and now I just I do not like looking at myself whatsoever.* (Rylie, WN-ILP, pre-int.)
Internalization of beauty standards	*I think cultural expectations of feminine looks. I think the pressure of being a supermom after you have kids; you are still supposed to like a certain way, but yet at the same time, having the resources – mainly time to do that – really significantly changes. And I think it’s physically, mentally, emotionally harder to reach that ideal or attain that ideal the older you get. Especially in the middle-of-age-life, where you have a lot of different pressures put on you to do different things.* (Lilly, WN-ILP, pre-int.)
Self-care	
Behaviors and thoughts	*It’s kind of weird, because it’s not that I’ve lost any weight because my eating has not really changed too much. But feeling good about myself and my body and just moving it and noticing throughout my days that the more I move my body in different ways, the better it is throughout my day, just doing anything in general. I’ve really gained an appreciation of that and just noticing when I get stronger.* (Bethany, WI-IE, pre-int.)
Body trust	*Just trusting my body. So I think if I can maybe eat better, maybe I’ll have more energy and then maybe I’ll feel better/find ways. … If I can feel better in my body, I think we’ll feel better.* (Poppy, WN-ILP, post-int.)

pre- or post-int. = pre- or post-intervention.

## Discussion

In this study, we evaluated the effects of an 8-week weight-inclusive Intuitive Eating-based course (WI-IE) on disordered eating, weight bias internalization, weight bias, and body image in adults working in a university setting compared to 8 weeks of a weight-normative Intensive Lifestyle Program (WN-ILP). The WI-IE group was associated with reductions in disordered eating from pre- to post-intervention when compared to the WN-ILP group. The WI-IE group experienced associations with greater improvements in IE than the WN-ILP group with medium effect, although these differences were not statistically significant. To expand on quantitative results from surveys, we also examined qualitative results from semi-structured interviews.

### Disordered eating and intuitive eating

Decreases in disordered eating IE and improvements in IE levels in the WI-IE group across time aligned with previous studies on IE-based interventions (Beintner et al., 2019; Burnette and Mazzeo, 2020; Burnette et al., 2022; Cole and Horacek, 2010; Rochefort et al., 2021). When examining the interviews to further explain and expand on these findings, Nancy from the WI-IE group shared about her shift from the restrict-binge cycle to feeling more relaxed around food in her post-intervention interview:

I'm not restricting or anything anymore. I was doing no sugar, no flour with the last thing I was doing, and feeling very deprived. So I'm not restricting and in that sense, it's like I said: it's showing that I'm not binging either, but neither am I not doing without. I have better times than others, but for the most part it’s much improved all the way around.

Similarly, Charlotte from the WI-IE group shared in her post-intervention interview that she experienced reduced guilt around certain foods she enjoys while also acknowledging her body’s hunger and fullness signals:

…it's opened up a lot and it's opened up the ability for me to say, ‘You know what, I'm not really hungry right now.’ Just because it's noon doesn't mean I have to eat. But if I want to, I can. I need to get what I need to fuel my body and not feel guilty about the fact that it was a carb or whatever.

Nancy and Charlotte’s experiences with escaping the restrict-binge cycle and reducing guilt around food aligns with findings in other IE interventions. For example, Burnette and Mazzeo’s (2020) study in college-age women found that both group-based and guided self-help IE interventions were associated with reductions in disordered eating behaviors like restriction, bingeing, and food-related guilt for up to 4 months post-intervention. Beintner et al. (2019) found that restrictive eating improved over 12 months of follow-up post-IE intervention. While our intervention did not have a follow-up period, our findings indicated similar short-term improvements, though these changes were not statistically significant and thus we cannot ensure if these changes were from the IE intervention itself, from being in a general support group with others seeking to heal their relationships with food, or something else.

Several researchers have previously noted that weight-centric health promotion and practices may worsen disordered eating behaviors (Mauldin et al., 2022; Romano et al., 2018). In this study, WN-ILP group participants did not significantly worsen in disordered eating thoughts and patterns, unlike previous findings on weight normative interventions (Leong et al., 2016; Richmond et al., 2020). This may be related to the fact that the WN-ILP instructors taught about the importance of attending to hunger and fullness cues to guide eating timing and amounts from a weight-normative perspective. While the authors of the intuitive eating book describe the concept as weight-inclusive (Tribole and Resch, 2020), the weight loss industry often adopts intuitive eating principles as a weight management strategy. Weight-normative approaches are likely not as effective in reducing disordered eating and improving intuitive eating as a fully weight-inclusive approach (Bruce and Ricciardelli, 2016), given that the emphasis of weight-normative programming is often on reducing portions or specific palatable foods. Teaching both calorie counting (external cues) and hunger and fullness (internal cues) may also present participants with increased cognitive dissonance and confusion around what are considered adaptive versus maladaptive eating behaviors. For example, Rylie from the WN-ILP group shared in her post-intervention interview:

I guess times where I get stressed out and binge eat and stuff, I definitely feel more guilty. I think that's more because I weighed myself and realized I gained weight last night. Guilt and shame from that kind of stuff and I spiraled a little bit last night. But I think that really only happens when I weigh myself when I feel that way?

Rylie realized she does not like feeling guilt and shame around food while eating, and that binge eating and weighing herself can bring about these feelings, which can lend itself to more binge eating. Weight maintenance or weight loss is a common mechanism that many health promotion programs use as a means to improve health (Kahathuduwa et al., 2016; Rothblum, 2018), and Rylie’s comments demonstrate cognitive dissonance—a cognitive conflict that happens when beliefs and actions do not align—towards commonly-held conventional health advice versus her personal experience of guilt, shame, and increased binge eating when regularly weighing herself. Rylie’s guilt and shame towards food was commonly shared among many WN-ILP participants, and aligns with patterns documented in the literature (Bottera et al., 2020; Romano et al., 2018). In fact, Bottera et al. (2020) found that feeling weight- or body-related shame is associated with binge eating. In another study, young adults who engaged in frequent self-weighing were more likely to gain weight, especially those who engaged in binge eating (Romano et al., 2018).

Cognitive dissonance also manifested with IE misconceptions that participants shared. IE misconceptions held steady among several WN-ILP participants. For example, Haley from the WN-ILP group shared in her post-intervention interview that she was enjoying eating more mindfully. She then followed up her reflections on mindful eating with:

I started reading about the 8-hour diet where you fast for so long and then eat for so long. So, looking into that, it was, “This doesn’t seem like this could be very healthy.” But, in actually researching it, the timeframes that you eat, they still also specify certain things that are considered healthy or power foods or different things that work towards your body to help stimulate your immune system or to help stimulate your metabolism or do these certain things.

Haley displayed understanding of listening to her body’s internal cues—which aligns well with weight-inclusive approaches—quickly followed up with a desire to engage in externally-driven forms of eating (e.g., time-bound, regardless of hunger or fullness signals) such as intermittent fasting. Haley shared later in her post-intervention interview her frustration with plateauing in many weight loss attempts, which may explain her desire to engage in a weight-centric approach to eating after trying to eat more mindfully.

In contrast, participants in the WI-IE class had more IE misconceptions at the beginning rather than the end of the course, which logically follows as they were actively learning and practicing IE principles throughout the course. For example, Bethany shared at the beginning of the course that she wanted to learn how to eat intuitively to stop “falling for cravings”:

But it's the cravings that keep hitting me so strongly that I know I need to do something more and it can be tricky sometimes because working out you can actually get hungrier and if you don't have the right food after you are working out, it's so easy to just fall for those cravings.

Eating intuitively is not about restricting oneself, but about learning how to listen to your cues, including honoring cravings (Tribole and Resch, 2020). Learning intuitive eating in a fully weight-inclusive manner can counter guilt and shame-fueled cycles by changing the focus away from a restrictive eating mindset towards a permission mindset (Tribole and Resch, 2017, 2020). The WI-IE participants learned how to be mindful during the eating experience, listen to internal cues, and accept cravings, without focusing on weight changes. After the WI-IE course, Bethany shared evolving feelings around previous IE misconceptions:

I think I do notice that it's easy to go back to old habits of eating quickly, eating while I'm doing something else, especially at work. I'm noticing a lot of these things, but I'm still having that thought of like, “Okay, this is what I'm doing.” And acknowledging it. So, I think in that sense, it's making some changes.

This phenomenon of shifting from guilt and shame-based eating towards permission to enjoy all foods aligns with the IE approach and is associated with reductions in disordered eating patterns (Burnette and Mazzeo, 2020; Tribole and Resch, 2020). Craven and Fekete (2019) found that those in their study who felt more weight-related guilt or shame had higher levels of binge eating, while IE moderated the relationship between shame and binge eating. This indicates that IE can help mitigate and dismantle feelings of shame, which can lead to deeper self-acceptance, as suggested by our participant Bethany.

### Body image

Associations with improvements in body appreciation and decreases in weight and shape concerns among WI-IE participants in this study align with Burnette and Mazzeo’s (2020) pilot study of a guided self-help and group-based IE course, as well as numerous other IE-based studies (Koller et al., 2020; Oswald et al., 2017; Ramos et al., 2022; Tylka and Wood-Barcalow, 2015). However, these improvements did not differ significantly across groups at the α < 0.007 level, despite the medium effect size.

While previous research indicated weight-normative health services can negatively impact body image (Richmond et al., 2020; Linardon and Mitchell, 2017), the WN-ILP group did not experience worsening body appreciation or weight/shape concerns. This may partially be explained by Hunger et al.’ (2015) Weight-Based Social Identity Threat model, which suggests that those who experience weight stigma have increased motivation to escape stigma by engaging in weight loss behaviors. Applying Hunger et al.’s (2015) model to the WN-ILP group, WN-ILP participants may have been actively pursuing weight loss behaviors to escape stigma, and thus experienced feeling body image-related relief. Lasikiewicz et al. (2014) conducted a systematic review on psychological improvements in weight loss interventions and found that such interventions temporarily improved body image regardless of whether or not individuals lost weight, which may also help explain the WN-ILP group’s results.

Moreover, the WN-ILP group had higher base-levels of body appreciation and lower base-levels of weight and shape concern than the WI-IE group. In addition, the WN-ILP intervention included a session on self-compassion, which may have had a protective effect on body image, as self-compassion is strongly associated with improved body appreciation (Linardon and Mitchell, 2017; Kelly and Stephen, 2016). The exercise component in the WN-ILP program could also be a reason why there were improvements in body appreciation and reductions in weight and shape concerns in both groups. Exercise was included as its own component in the WN-ILP program, while the WI-IE program encouraged joyful movement but did not have an explicit piece of the course dedicated to physical activity. Physical activity is shown to boost self-confidence and improve body image, regardless of changes in body weight, shape or size (Davis and Cowles, 1991; Gilani and Dashipour, 2016; Schoenefeld and Webb, 2013), and WN-ILP group members met with an exercise physiologist outside of the WN-ILP class time to set goals around engaging in more physical activity. This structured exercise component in the WN-ILP class, which was not part of the WN-IE intervention, may also explain why body image improved in both groups.

While both groups experienced improvements in body image constructs, the WI-IE group had greater mean improvements in body image constructs compared to the WN-ILP group, although the differences were not significant at the α < 0.007 level. Nancy from the WI-IE group shared that post-intervention:

It [body image] fluctuates in-between…because there's still times that I will look at myself harshly, but, but overall, I feel good about what my body has done for me and where I'm at because I think that I am a harsher judge on myself than other people are looking at me.

Nancy found that while she still views her body negatively at times, she shifted to feeling good about her body and focusing on function over form (Shang et al., 2021). It was apparent that several WI-IE group participants had tried shifting their focus from their bodies’ form (aesthetic appeal) to their function:

…it's funny because usually when you think about exercise, the reward is to physically look great, to lose weight. But actually, when I go to this small gym, they offer HIIT classes, and there's all sorts of different people in the class. So, it's just been really great to think, ‘Well, these are just people wanting to get healthy and still be able to do the things that they like to do.’ It's been a different exercise environment where it's about feeling the best that you can feel, not necessarily for an outward appearance of looking great and that sort of thing (Bethany, WI-IE, pre-intervention interview).

Nancy and Bethany’s responses suggest that finding peace in focusing on function over form and enjoying their bodies’ capabilities feels important. They each named intrinsic sources of motivation, which aligns with the IE approach (Tribole and Resch, 2020). Discovering sources of motivation that are not weight-related may be impactful in supporting long-term engagement with physical activity (Hartmann et al., 2015). Bruce and Ricciardelli (2016) conducted a meta-analysis reviewing 24 cross-sectional IE studies and found that IE consistently predicted body appreciation and satisfaction, better emotional functioning, unconditional self-regard and optimism, and greater motivation to exercise.

### Weight bias internalization (WBI) and weight bias

This study did not show statistically significant changes in WBI and weight bias across time and groups. Previous research indicates that WBI has been successfully addressed with media literacy and “rejecting the thin ideal” education (Kite and Kite, 2021; Koller et al., 2020), of which neither course in this study focused on extensively. The lack of statistically significant decreases in WBI and weight bias may be due to the very brief intervention, as WBI can become deeply entrenched in one’s identity (Decker et al., 2018; Pearl and Puhl, 2018; Pudney et al., 2020; Stevens and Griffiths, 2020). Another possible explanation for the lack of statistically significant decreases in WBI and weight bias is that participants in the WI-IE group had initially higher levels of WBI and lower levels of IE compared to the WN-ILP group. Those who seek out weight-inclusive care may have more negative perceptions towards themselves and lower self-confidence in their ability to listen to their bodies (Tylka et al., 2014). Charlotte from the WI-IE group illustrated this when she shared in her pre-intervention interview:

…My mom would tell me, you maybe shouldn't eat that or maybe let’s just go for a walk. You need to be moving and you need to go exercise and you need to go for a walk. And then she put me on a diet, a pseudo-Weight Watchers thing where I was supposed to keep track of what I ate during the day, and those kinds of things.

In contrast, Stephany from the WN-ILP group demonstrated lower initial WBI and higher IE levels than Charlotte and shared in her pre-intervention interview that she grew up in a family dynamic where dieting was not pushed on her at any point in her childhood:

My family was also super… they just didn't care. We would always go out and be outside and traveling and doing stuff. But it wasn't ever…no one was ever putting pressure on people or talking about diets and stuff. I don't know, I just feel like I grew up in a pretty neutral environment, so I never really thought about it when I was a little.

Charlotte and Stephany’s contrasting childhood upbringings and family dynamics regarding weight and body likely impacted the program they chose to enroll in later in life. Stephany had few negative perceptions around her body, while Charlotte held deep guilt and shame about her body, shaped from familial pressure to conform to beauty ideals. In a longitudinal study by Simone et al. (2021), higher levels of parental weight talk in young adult women was associated with the highest levels of extreme unhealthy weight control behaviors (e.g., compulsive exercise, caloric restriction, bingeing, purging), implying negative body perceptions among those who had parents persistently talk about weight during their childhoods. In another study, maternal attitudes towards weight, especially fear of getting fat (fatphobia), significantly correlated with their daughters’ internalized weight bias and thin-ideal internalization (Gagnon-Girouard et al., 2020). Both of these studies support our quantitative and qualitative results; namely, that family history of weight talk can lead to increased pressure to conform to a body ideal, which can lead to unhealthy behaviors over time and ultimately lead those struggling with body image and unhealthy weight control behaviors to seek out a different line of treatment.

## Strengths, limitations, and future directions

This study was one of the first to employ a worksite wellness weight-inclusive IE health promotion course based on all 10 IE principles and approved by the founders of the IE framework, Evelyn Tribole and Elyse Resch. This study was also one of the first to test the effectiveness of a weight-inclusive IE course compared to a weight-normative comparison group. Additionally, the majority of previous studies on IE utilized quantitative or mixed-methods designs with short-answer open-ended questions (Burnette and Mazzeo, 2020; Burnette et al., 2022; Cole and Horacek, 2010), while this study utilized an explanatory sequential mixed-methods design with longer, semi-structured interviews that gave deeper insight into the quantitative results. While often seen as a limitation for participants to self-select the course instead of being randomized into the course, this naturalistic setting allowed us to observe the personalities and life circumstances that draw participants to a specific employee wellness course, be it weight-inclusive or weight-normative.

As far as limitations, this study had a small total sample size (*n* = 42) that was predominately college-educated, white females with a short intervention design (8 weeks) and no follow-up period to see if changes in the intervention group persisted several months or years post-intervention. In addition, participants self-selected which course they wanted to join rather than being randomized into a course, which prevented us from making causal inferences with our data. Moreover, the WI-IE focused heavily on the food/body/exercise relationship, while WN-ILP covered a broader spectrum of topics (e.g., stress, nutrition, PERMA, etc.). The difference in outcomes may reflect content focus and intensity and not necessarily the weight-inclusive philosophy. As for instruments, the baseline IES-2 Cronbach’s alpha for reliability was low (0.60), and thus limits the ability to detect true change. Finally, there were uneven sample sizes across groups (*n_WN-IE_* = 26, *n_WN-ILP_* = 16) due to one more section of the WI-IE course being offered, and thus higher enrollment to recruit from in the WI-IE group compared to the WN-ILP group. Finally, while overall program retention was high (95% in the WI-IE group and 92% in the WN-ILP group), WI-IE study attrition was quite high with only 26 of the 34 WI-IE participants enrolled in the study completing both pre- and post-course surveys, compared to 16 of 17 of the WN-ILP participants completing both pre- and post-course surveys. This may be due to WI-IE participants’ sensitive relationships with food, body, and exercise.

Future research should focus on recruiting a more representative sample with more racial, ethnic, gender, and socioeconomic diversity, and randomizing participants into their courses or utilizing a waitlist control group design. Future research would also benefit from longer follow-up, ideally spanning three or more years, to examine how health behaviors impact health outcomes long-term (Hall and Kahan, 2018; Selensky and Carels, 2021; Selivanova and Cramm, 2014).

## Conclusion

In this study, we found significant associations with improvements in disordered eating in the weight-inclusive group compared to the weight-normative group. However, changes in weight bias, weight and shape concerns, and body appreciation did not differ significantly between the two groups. Interviews showed the weight-normative group had generally different goals than the participants in the weight-inclusive group. Based on interviews and quantitative data analysis, it seems that those who struggled more with food, body, and movement chose the WI-IE course. This suggests that people who are drawn to weight-inclusive health promotion may have a more fraught relationship with food than those who are not drawn to weight-inclusive programming. Importantly, this study did not measure nor aim for weight loss. This study highlights key differences between the two types of programs and supports the need for future randomized-controlled trials to further examine these findings and improve wellness program offerings.

## Data Availability

The anonymized data supporting the conclusions of this article will be made available by the authors upon request.
