# Supplementary material for: Weight-inclusive intuitive eating course associated with reductions in disordered eating in adults: a mixed methods study
Source: Front Psychol. 2026 Oct 6;17:1953974. doi: 10.3389/fpsyg.2026.1953974 (PMC13680151; doi:10.3389/fpsyg.2026.1953974)
Supplement: Supplementary file 1 [file Supplementary_file_1.DOCX]

**Appendix A**: Semi-Structured Interview Question Guide

Interviewer:

Do you consent to letting your answers in this interview be used anonymously as data for a

research project? (Verbal consent)

[  ] Yes

[  ] No

*If no, discontinue interview*

This interview will take approximately 15-30 minutes.

What would you like your pseudonym for transcription to be? __________________

*If no preference, create pseudonym for interviewee*

**Semi-Structured Interview Questions**

*Please answer the following questions as openly and honestly as you can.*

1. Do you consider yourself to have positive, poor, or “somewhere-in-between” body image?

a) Why or why not? What has contributed to your view(s) around your body? (e.g., family,

media, work culture)

2. How would you describe your current relationship with food, body, and movement?

a) How does this relationship affect your quality-of-life?

3. What do you think would be most beneficial for you to develop better body image and/or a healthy relationship with food?

4. What do you most hope happens during the IE (or ILP) course?

a) What are you most excited to learn about and/or practice?

5. Post only: Were your expectations met during the IE (or ILP) course? Please describe.

a) What impacted you most during the IE (or ILP) course?

b) Has your relationship with food, body, and movement changed? If yes, how has it

changed? How has this shift affected your quality of life?

Thank you for participating in this interview! I hope your program goes well for you. [Post-only: I wish you the best!]
